# Incorporating Power-Law Model and ERA-5 Data for InSAR Tropospheric Delay Correction Analysis

**DOI:** 10.3390/s25030716

**Published:** 2025-01-24

**Authors:** Dongxu Huang, Junyu Wang, Menghua Li, Cheng Huang, Bo-Hui Tang

**Affiliations:** 1 Faculty of Land Resources Engineering, Kunming University of Science and Technology, Kunming 650093, Chinatangbh@kust.edu.cn (B.-H.T.); 2 Natural Resources and Planning Bureau of Honghe Hani and Yi Autonomous Prefecture, Mengzi 661000, China; 3 Yunnan International Joint Laboratory for Integrated Sky-Ground Intelligent Monitoring of Mountain Hazards, Kunming 650093, China; 4 Yunnan Key Laboratory of Quantitative Remote Sensing, Kunming 650093, China; 5 Yunnan Institute of Geological Environment Monitoring, Kunming 650216, China

**Keywords:** InSAR, tropospheric delay correction, ERA-5, power-law model

## Abstract

InSAR technology effectively monitors urban subsidence and evaluates the stability of infrastructure across extensive regions. Atmospheric tropospheric delay constitutes a significant source of error that adversely impacts the accuracy of InSAR deformation measurements. The meteorological conditions in the highland basin region are complex, and there is a notable deficiency of sufficient sounding balloon observations. This study replaces the sounding balloon data in the power-law model with ERA-5 data (PLE5) to correct the InSAR atmosphere phase delay. This method was tested in Dali utilizing Sentinel-1 data. By comparing its performance against the GACOS model, traditional linear model, and ERA-5 correction, the PLE5 model exhibited the lowest phase standard deviation. This method provides an alternative approach for applying the power-law model in regions with limited sounding balloon data, enhancing the accuracy of InSAR tropospheric delay correction.

## 1. Introduction

Interferometric Synthetic Aperture Radar (InSAR) is a powerful tool capable of monitoring surface deformation with sub-centimeter precision at spatial resolutions from decimeters to tens of meters [1,2]. It has been demonstrated as an effective tool in the application of earthquake [3], land subsidence [4], landslide monitoring [5], and the stability of infrastructure [6], which provides a choice for surveying large-area ground displacement and analyzing the underlying mechanisms of surface displacements for disaster prevention and mitigation.

Satellite radar signals encounter atmospheric delays as they propagate through the Earth’s atmosphere, rendering atmospheric errors one of the most significant sources of distortion, which can introduce considerable noise and obscure the deformation phase. The atmospheric disturbances consist of both ionospheric and tropospheric components [7]. The influence of the ionosphere depends on the total electron content (TEC) and primarily impacts long-wavelength signals [8]. The ionospheric effect on InSAR can be mitigated by selecting a reference SAR image with the minimum rate of TEC Index and then estimating the differential ionospheric TEC using the range split-spectrum interferometry method and the International Reference Ionospheric model [9]. Meanwhile, tropospheric delays are nondispersive effects affected by pressure, temperature, and humidity [10]. Considerable attention has been devoted to mitigating the effects of tropospheric delays in recent years, though achieving effective mitigation remains challenging [11].

The tropospheric delay that is comprised of hydrostatic delay and wet delay [12,13] can often be modelled as a mixture of vertically stratified and turbulent components in InSAR applications [10]. Currently, several studies have considered the turbulent atmospheric delay component to be spatially and temporally random, which can be mitigated by temporal filtering of large time series acquired by synthetic aperture radar (SAR) [14,15,16,17]. On the other hand, when using stacking or more complicated time series methods, stratified tropospheric delays can produce long time-series biases in strain rate estimates when the timing of seasonal oscillations is not adequately sampled [18].

Currently, there are two main categories of tropospheric delay correction methods used in InSAR research. The first category is the predictive approach that atmospheric correction methods based on external data, such as the Global Navigation Satellite System (GNSS) [19,20,21], Numerical Weather Models (NWMs) [17,22], and Moderate Resolution Imaging Spectroradiometer (MODIS) data [23], can provide complete tropospheric delay information. However, the spatial and temporal resolution differences between interferograms and external data restrict this method [24]. Thus, based on ERA-I reanalysis, a new method for estimating tropospheric delays in radar interferograms using a power-law model is proposed [25]. This method attenuates the effects of the power-law model sounding data and the spatial and temporal resolution of using external data while uniting the power-law model with external data to improve the accuracy of the atmospheric delay correction.

The second category is the empirical method, which corrects the phase delay within the interferometric phase itself by modelling the relationship between phase and topography and using known surface elevation information to estimate the tropospheric delayed phase for correction purpose [26]. This methodology comprises two models, a linear function model and a power-law function model, which estimate the effect of tropospheric delay on the interferogram by analyzing the linear relationship or exponential relationship between the interferometric phase and the terrain elevation. The power-law function model accounts for spatial variability and can be applied across various temporal, atmospheric, and deformation conditions [24]. Estimation of parameters in the conventional power-law model requires sounding data, but the determination of power-law coefficients is often constrained by the limited availability of such data in many areas. Additionally, sounding data may contain discontinuities, such as jumps, due to changes in instrumentation, observing practices, or station locations [25].

In this study, we propose a power-law model incorporating data from the ERA-5 dataset (PLE5) to correct the InSAR tropospheric delay. This approach involves fusing the ERA-5 reanalysis dataset to estimate the coefficients of the power-law model for each SAR acquisition, rather than using sounding data. The ERA-5 reanalysis products, obtained from the European Centre for Medium-Range Weather Forecasting (ECMWF), have been reported to be more accurate and applicable in capturing atmospheric water vapor than their predecessor, ERA-I [27,28,29,30,31]. We compared the PLE5 model approach with the commonly used empirical linear correction model, the GACOS correction model, and ERA-5 dataset correction method to evaluate its effectiveness and applicability in InSAR tropospheric delay correction.

## 2. Study Area and Data Processing

### 2.1. Study Area and Dataset

Dali City, Yunnan Province, is situated in the western region of Yunnan Province. The topography is high in the northwest and low in the southeast, with the Hengduan Mountain Range west of Erhai Lake exhibiting significant topographic variation. The Central Yunnan Plateau, located east of Erhai Lake, features an elevation range of 1386 to 4122 m, characteristic of a subtropical plateau monsoon climate. The proximity to Erhai Lake and the prevalence of monsoon circulation lead to variations in temperature, wind speed, wind direction, and surface humidity, which, in turn, affect the structure of the atmospheric boundary layer [32]. SAR imaging is significantly affected by vertical stratification delays in the troposphere, making the correction of tropospheric delays crucial in InSAR applications.

**Figure 1:**
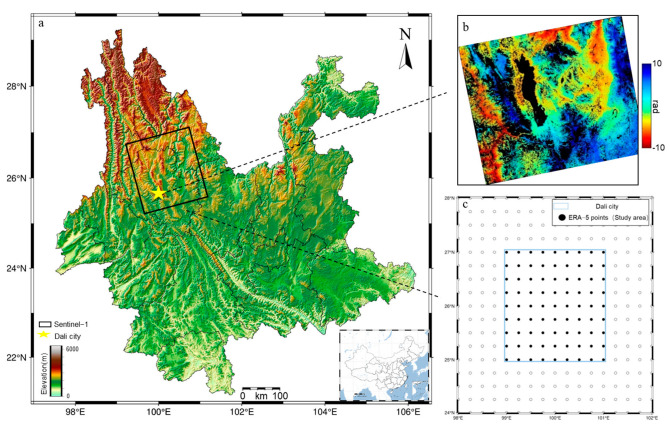
(**a**) Topographic map of Yunnan Province; the base map of this study area is the AW3D30 DEM of Dali City. (**b**) Original differential interferogram without atmospheric correction and (**c**) distribution of grid point locations of the ERA-5 dataset in Dali City.

The Sentinel-1 satellite has a short revisit cycle (12 days) and provides freely available observational data for both ascending and descending orbits. A total of 96 scenes of C-band Sentinel-1A ascending data, spanning from 7 January 2019, to 28 March 2022, were acquired from the European Space Agency (ESA) for Dali, Yunnan Province, at 19:24 local time (11:24 UTC). The data processing utilizes ALOS World 3D data of Dali, Yunnan Province, with a resolution of 30 m (see Figure 1a) [33]. The SAR data were interferometrically preprocessed to generate raw interferograms (e.g., Figure 1b) that include atmospheric effects, after denoising and phase unwrapping.

**Figure 2:**
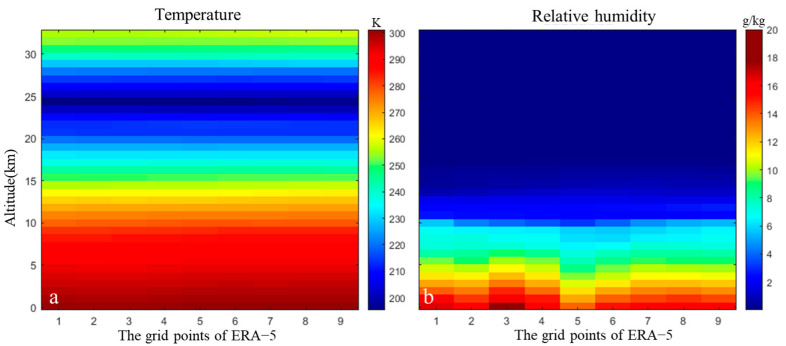
Vertical contour plot of temperature at 9 grid points along 25° N from ERA-5 data, showing (**a**) temperature variation with altitude and (**b**) relative humidity variation with altitude.

The ERA-5 dataset from the ECMWF (see Figure 1c) was used to retrieve barometric pressure, temperature, relative humidity, and other key meteorological elements for Dali City (25° N–27° N, 99° E–101° E) for the period 2019 to 2022. These data were employed to investigate the spatial distribution of tropospheric delays and assess the impact of corrections. We extracted the vertical contours of temperature and humidity ratios from the acquired ERA-5 data, as illustrated in Figure 2. The vertical contour plot of temperature (see Figure 2a) indicates that temperature decreases with increasing altitude until a certain elevation is reached, after which it begins to increase. The vertical contour plot of relative humidity (see Figure 2b) demonstrates that relative humidity consistently decreases with increasing altitude, approaching zero as barometric pressure reaches 200 hPa. This observation indicates that the water vapor content in the area is concentrated below an elevation of 12 km.

### 2.2. InSAR Processing

**Figure 3:**
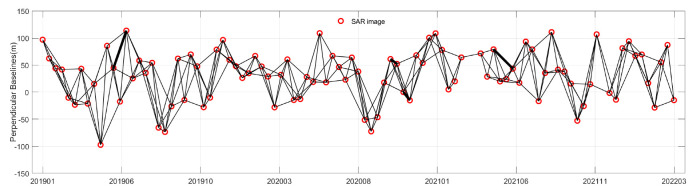
Spatial and temporal baselines map of the interferogram. Each red dot represents an SAR acquisition and each line represents an interferogram.

We utilized the Gamma tool in the StaMPS 4.1 software to preprocess the acquired SAR images interferometrically, resulting in the generation of 258 interferograms, the spatial baseline distribution of which is depicted in Figure 3. Subsequently, we employed the COP-DEM data to eliminate the flat-earth effect, utilized the precision orbital data records (ODR) provided by ESA to correct for orbital errors, and applied the Goldstein filtering method to process the interferograms. Finally, we utilized the SNAPHU tool for phase unwrapping of the interferograms [34], yielding interferograms that have not been corrected for tropospheric delay. Later, we verified the applicability of the PLE5 method based on four of the processed interferograms, with the relevant parameters presented in Table 1.

## 3. Methodology

### 3.1. Correcting Tropospheric Delays with the ERA-5 Reanalysis Dataset

The spatial heterogeneity of the troposphere causes the atmospheric refractive index to change in response to variations in meteorological elements such as temperature, barometric pressure, and relative humidity. According to the spatial variation model of the atmospheric refractive index, the tropospheric delay model in the line-of-sight direction can be expressed as follows:

(1) $$\Delta d_{LOS}^{hydr}(h)=\frac{10^{-6}}{\cos\theta }\int_{h}^{h_{top}}K_{1}\frac{P(h)}{T(h)}dh$$

(2) $$\Delta d_{LOS}^{wet}(h)=\frac{10^{-6}}{\cos\theta }\int_{h}^{h_{top}}[(K_{2}-\frac{R_{d}}{R_{v}})\frac{e(h)}{T(h)}+K_{3}\frac{e(h)}{T{\left(h\right)}^{2}}]dh$$

(3) $$\Delta d_{LOS}(h)=\Delta d_{LOS}^{hydr}(h)+\Delta d_{LOS}^{wet}(h)$$

where $\Delta d_{LOS}\left(h\right)$ denotes the total tropospheric delay in the line of sight, $\Delta d_{LOS}^{hydr}\left(h\right)$ represents the hydrostatic delay, $\Delta d_{LOS}^{wet}\left(h\right)$ represents the wet delay, and *θ* denotes the angle of incidence of the radar signal. The constants are defined as follows: $K_{1}=77.6\;{\mathrm{Khpa}}^{-1}$, $K_{2}=71.6\;{\mathrm{Khpa}}^{-1}$, and $K_{3}=3.75\times 10^{5}\;K^{2}{\mathrm{hpa}}^{-1}$ [25,35]. Additionally, *P* denotes barometric pressure, *T* denotes temperature, and *e* denotes partial pressure of water vapor. The gas constants for dry air and water vapor are $R_{d}=287.05\;{\mathrm{Jkg}}^{-1}K^{-1}$ and $R_{v}=461.495\;{\mathrm{Jkg}}^{-1}K^{-1}$ [25,36]. The value of *e* is estimated using the relative humidity provided by the ERA-5 reanalysis dataset, calculated via the Clapeyron–Clausius equation as follows [25,37]:

(4) $$e\left(h\right)=e^{*}\left(h\right)\frac{R_{e}\left(h\right)}{100}$$

(5) $$e_{w}^{*}\left(h\right)=a_{1}e^{a_{3,w}\frac{T\left(h\right)-T_{o}}{T\left(h\right)-a_{4,w}}},T\left(h\right)>T_{0}$$

(6) $$e_{i}^{*}\left(h\right)=a_{1}e^{a_{3,i}\frac{T\left(h\right)-T_{o}}{T\left(h\right)-a_{4,i}}},T\left(h\right)<T_{i}$$

(7) $$e^{*}\left(h\right)=e_{i}^{*}\left(h\right)+{\left(e_{w}^{*}\left(h\right)-e_{i}^{*}\left(h\right)\right)}^{e^{\frac{T\left(h\right)-T_{i}^{2}}{T_{0}-T_{i}}}},\;T_{0}<T\left(h\right)<T_{i}$$

where $R_{e}\left(h\right)$ denotes relative humidity and $e^{*}\left(h\right)$ denotes the saturated water vapor partial pressure, $e_{w}^{*}\left(h\right)$ denotes the partial pressure of supersaturated liquid water, and $e_{i}^{*}\left(h\right)$ denotes partial pressure of supersaturated ice. The constants are defined as follows: $T_{0}$ = 273.16 K, $T_{i}$ = 250.16 K, $a_{1}$ = 611.21 hPa, $a_{3,w}$ = 17.502, $a_{4,w}$ = 32.19 K, $a_{3,i}$ = 22.587, and $a_{4,i}$ = −0.7 K.

### 3.2. Correcting Tropospheric Delays with PLE5 Model

The power-law function model is founded on the relationship of power-law distribution between the tropospheric delay phase and surface elevation. Subsequently, the power-law index is calculated, and the model is fitted accordingly. The power-law relationship can be expressed as:

(8) $$\Delta \varphi_{tropo}=K^{\prime }{\left(h_{0}-h\right)}^{\alpha }+\Delta \varphi_{c},h<h_{0}$$

where $\Delta \varphi_{tropo}$ represents the tropospheric delay of the interferogram, $K^{\prime }$ is the slope between tropospheric phase and surface elevation, *α* is the power-law decay coefficient estimated from the ERA-5 reanalysis data, and $\Delta \varphi_{c}$ is the phase of the tropospheric delay at a specific height $h=h_{0}$, which can also be expressed as the overall offset of the interferogram tropospheric delay. $h_{0}$ denotes the constraint elevation, representing the minimum height at which the absolute tropospheric delay equals zero.

**Figure 4:**
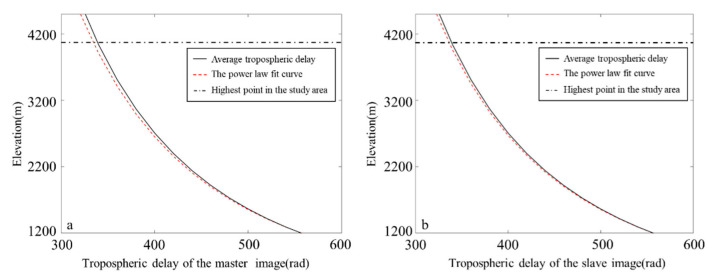
The relationship between the mean tropospheric delay and surface elevation for (**a**) the master image/20190107, and (**b**)the slave image/20201109.

To further validate the applicability of the PLE5 model for our study area, we fit to determine whether there is a power-law relationship between relative tropospheric delay and surface elevation for both the master and slave image during InSAR processing using the ERA-5 reanalysis dataset. As illustrated in Figure 4, a power-law distribution exists between the tropospheric delay and surface elevation. The black solid line represents the mean tropospheric delay obtained from the ERA-5 reanalysis dataset, which was obtained using the ERA-5 reanalysis dataset with time periods of 11:00 UTC and 12:00 UTC moments, the red dashed line shows the power-law curve of the tropospheric delay fitted to the surface elevation, and the black dashed line shows the highest point in the study area. There is a clear power-law relationship between the tropospheric delay and surface elevation for the master and slave images in Figure 4a,b, with the fitted power-law decay coefficient of 1.2 and 1.3, respectively.

The other images also calculate their respective power-law coefficients in turn. Based on the results of fitting the tropospheric delay to surface elevation in the study area, we found that the differences in the power-law decay coefficients for both the master and slave images are minimal. Thus, our study area can use the PLE5 model to simulate the tropospheric delay of the interferogram. The atmospheric refractive index varies across different images. Therefore, the mean value of the coefficients fitted using only the master and slave images does not accurately reflect the power-law relationship in each image. To mitigate this uncertainty, the power-law decay coefficient can be estimated from the fitted relationship between the relative tropospheric delay and surface elevation of the interferograms corresponding to the master and slave images. The relationship can be expressed as:

(9) $$\Delta \varphi_{tropo}=\Delta \varphi_{tropo}^{m}-\Delta \varphi_{tropo}^{s}$$

where $\;\Delta \varphi_{tropo}$ denotes the relative tropospheric delay of interferogram *m* and interferogram *s*, $\Delta \varphi_{tropo}^{m}$ and $\Delta \varphi_{tropo}^{s}$ represent the tropospheric delay for interferogram *m* and interferogram *s*, respectively, and $\Delta \varphi_{tropo}\;$ can be further written as:

(10) $$\Delta \varphi_{tropo}=K_{m-s}^{\prime }\;{\left(h_{0}-h\right)}^{\alpha },h<h_{0}$$

(11) $$K_{m-s}^{\prime }=K_{m}^{\prime }-K_{s}^{\prime }$$

**Figure 5:**
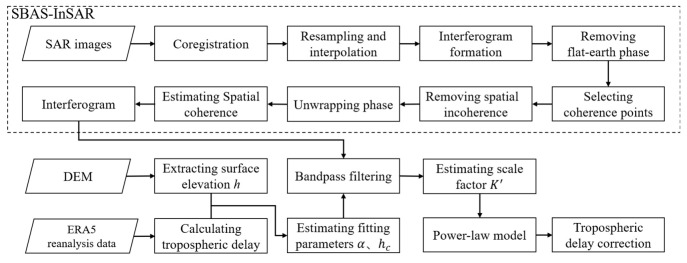
Workflow of PLE5 model for tropospheric delay correction in time-series InSAR.

Lastly, the workflow of the method is presented in Figure 5 and is as follows: the standardized InSAR technique employs the SBAS-InSAR algorithm to extract ground-based coherent point targets, followed by phase unwrapping and spatial coherence estimation. To enhance the accuracy of the analysis, a tropospheric delay correction module is integrated, utilizing the downloaded ERA-5 dataset to reanalyze temperature, barometric pressure, relative humidity, and potential height. The tropospheric delay phase is computed in accordance with Equations (1)–(3). The elevation *h* of the coherent point target is derived from external DEM data and both the power-law exponent and reference elevation are fitted usng a double-logarithmic method. Subsequently, band-pass filtering [24], which reduces noise and enhances key features for analysis, is applied to the interferogram phases, and the scale factor *K*’ is estimated to establish a power-law model, which is then utilized to calculate the tropospheric delay for the correction of InSAR results.

## 4. Results and Analysis

In this study, the PLE5 method is applied to InSAR tropospheric delay correction in Dali City, Yunnan Province, to verify its effectiveness in correcting tropospheric delay. Additionally, the method is compared and analyzed against conventional correction methods, including the empirical linear model, GACOS model, and ERA-5 reanalysis dataset, to evaluate its effectiveness and applicability in InSAR tropospheric delay correction.

### 4.1. Parameter Estimation for PLE5 Model

In the study of tropospheric delay correction using the PLE5 model, when parameter estimation is performed using sounding balloons, it is assumed that the power-law decay coefficient and reference elevation for each interferogram are invariant constants [24]. When employing ERA-I reanalysis data for parameter estimation, it is assumed that the power-law decay coefficient and constraint elevation are time-varying, resulting in different parameters for each interferogram [25]. Due to the sparse density of sounding balloon stations, which cannot cover the study area, and the inability to download their meteorological data after January 2020, we use the ERA-5 reanalysis dataset, an updated product of ERA-I with improved temporal and spatial resolution, transitioning from six-hourly to hourly updates, instead of sounding balloon data to estimate the parameters α and $h_{0}$.

**Figure 6:**
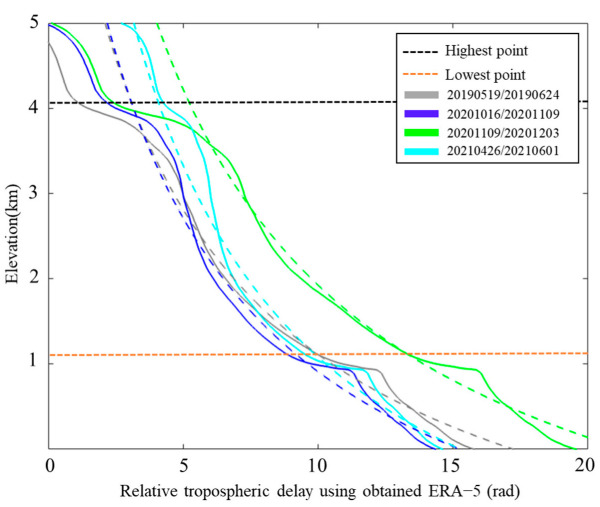
The distribution relationship between relative tropospheric delay and elevation of four interferograms.

The relative tropospheric delays obtained from the ERA-5 reanalysis dataset can be fitted to a power-law relationship with elevation, and the distribution law between relative tropospheric delay and elevation enables the fitting of the two required parameters, as shown in Figure 6. The results indicate that the power-law decay coefficients and reference elevations vary for four-scene interferograms. The gray dashed solid line indicates the interferogram 20190519/20190624, which is fitted with a power-law decay coefficient of 1.6 and a constrained elevation of about 6 km; the blue solid line indicates the interferogram 20201016/20201109, which is fitted with a power-law exponent of 1.1 and a reference elevation of about 5 km; the green-colored solid line indicates the interferogram 20201109/20201203, which has a fitted power-law decay coefficient of 1.5 and a constrained elevation of about 5 km; the cyan-colored solid line denotes the interferogram 20210426/20210601, which has a fitted power-law decay coefficient of 1.3 and a constrained elevation of about 4.8 km; and dashed lines denote the power-law fitted curve of each interferogram.

The remaining interferograms sequentially obtain the power-law model parameters using the described method, thereby determining the tropospheric delay for each. Analysis of the relationship between relative tropospheric delay and elevation reveals a power-law relationship in the study area. It is further confirmed that the PLE5 model can effectively analyze and mitigate tropospheric delays in interferograms, thereby reducing uncertainty in the results.

### 4.2. Estimated Tropospheric Delays

In this study, the tropospheric delay simulations for each interferogram were generated using the aforementioned PLE5 model, based on the parameters obtained in Section 4.1. The correction effectiveness of the PLE5 method was evaluated by comparing it with three widely used approaches: GACOS model correction, traditional linear model correction, and ERA-5 reanalysis dataset correction.

**Figure 7:**
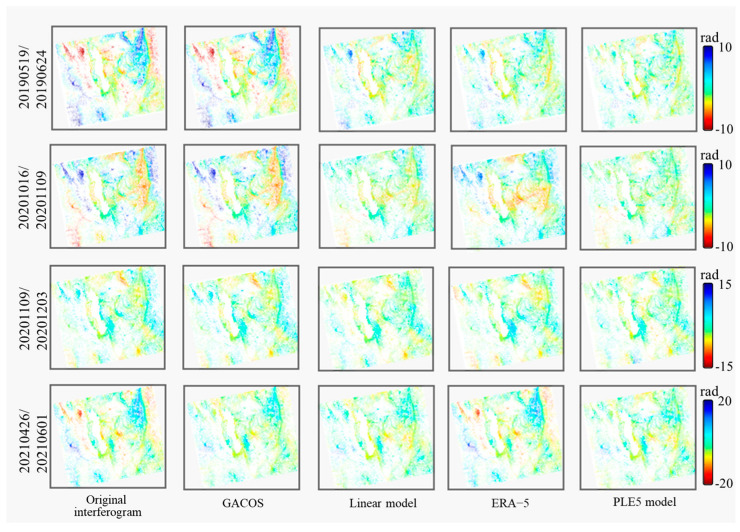
The unwrapped interferograms before and after tropospheric delay correction by four different methods: GACOS, traditional linear model, ERA-5 reanalysis dataset, and PLE5 model of four scenes.

A visual analysis of tropospheric delay and original interferogram delay simulated by different methods reveals the extent of atmospheric influence on InSAR and assesses the effectiveness of each model in estimating atmospheric delay. The GACOS and ERA-5 data corresponding to the times of the SAR images are downloaded to estimate the tropospheric delays. Figure 7 presents the results of interferograms before and after tropospheric delay correction, with the tropospheric delays estimated using the four different methods.

The phase of the original interferences varies drastically, with significant topography-related trend changes, and is, therefore, subject to severe atmospheric noise. After atmospheric corrections using the four models—GACOS, linear, ERA-5, and PLE5 model—residual tropospheric delays remain in certain regions of the interferograms, likely due to instances of overestimation or underestimation of the delay. Among the applied methods, the PLE5 model demonstrates effectiveness in mitigating tropospheric delays over large areas, providing reliable results for InSAR atmospheric corrections.

### 4.3. Comparing with Different Methods

**Figure 8:**
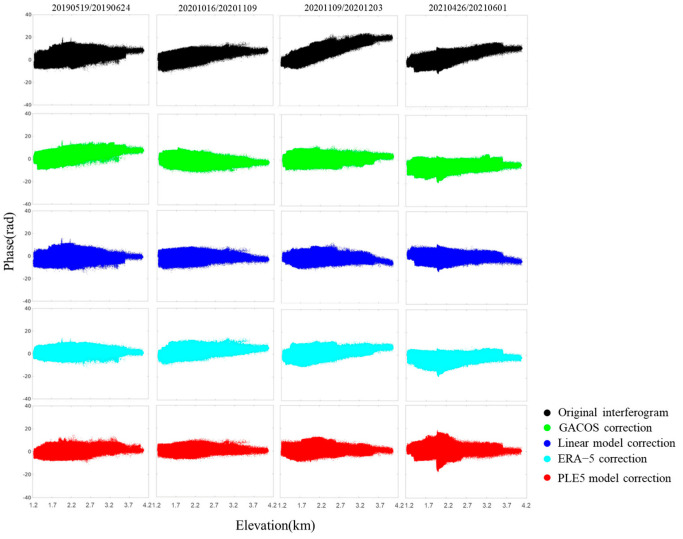
Comparison of the relationship between interferogram phase and elevation in the study area before and after correction by four different tropospheric delay methods.

After comparing the results of the four tropospheric delay correction methods with the original interferograms, the relationship between the interferograms before and after correction and elevation is analyzed in Figure 8. The black scatter plot in Figure 8 reveals a linear relationship between the original interferogram phase and elevation, indicating that the vertical stratification delay constitutes a significant portion of the tropospheric delay. After applying the four tropospheric delay correction methods, all show some effect in reducing the vertical stratification delay and the interferogram phase-to-elevation ratios are noticeably reduced. The red scatter plot in Figure 8 demonstrates that most of the phases after the PLE5 model correction stabilize around zero.

Although the four different correction methods show some effect, certain methods demonstrate limited effectiveness, with incomplete corrections or even the introduction of phase errors. The interferogram 20201109/20201203 in Figure 8 shows that the phase of the original interferogram (black scatter plot) exhibits an overall upward deviation and, while the phase (blue scatter) improves after correction using the traditional linear model, the phase value deviates downward, introducing a new error at elevations above 3700 m. This may be attributed to the fact that the conventional linear model uses only a scale factor constant to represent the phase–elevation relationship, which fails to account for the spatial variability of the tropospheric delay. The standard deviation magnitude is subsequently used to compare the correction effects of the different methods.

**Figure 9:**
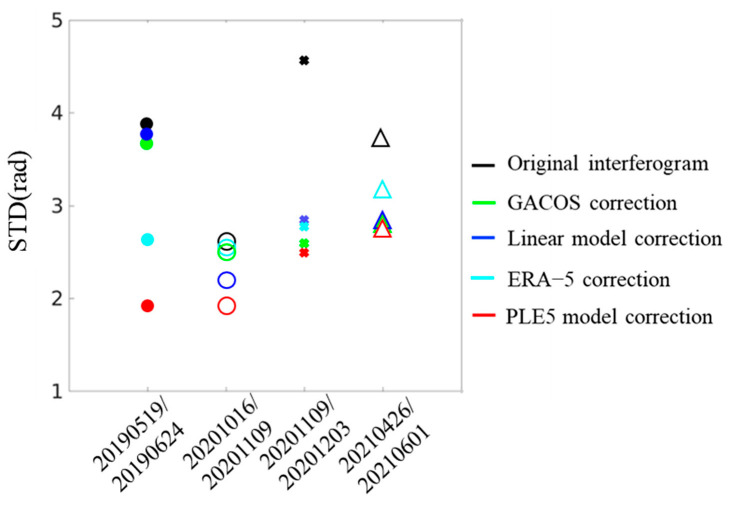
Comparison of the standard deviations of the original phases of four interferograms before and after correction by different methods for tropospheric delays.

The standard deviation (SD) is a crucial indicator of the effectiveness of atmospheric correction, particularly in estimating the impact of tropospheric delay correction in the absence of significant surface deformation and displacement [29]. By comparing the standard deviation of tropospheric delays corrected by different methods, we can determine which method is most effective in mitigating tropospheric delays. The standard deviation of the original phase of the four interferograms, after tropospheric delay correction by different methods, is shown in Figure 9. The results indicate that the standard deviation of the corrected phases for all four methods is smaller than that of the original interferogram phases. Among them, the PLE5 model, with the smallest standard deviation, proves to be the most effective.

**Figure 10:**
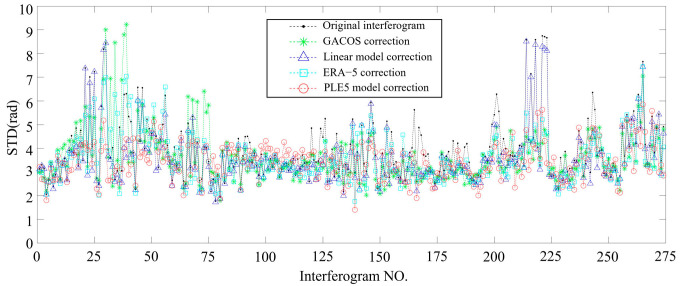
Variation in standard deviation of differential interferogram phase in the study area before and after correction by different methods of tropospheric delay.

As shown in Figure 10, the standard deviation changes of the phase in all differential interferograms in the study area, before and after tropospheric delay correction by different methods, indicate that most interferograms have been successfully corrected by the four methods, resulting in a reduction in the tropospheric delay and a corresponding decrease in phase standard deviation. However, as can be seen in the figure, the phase standard deviation in a small number of interferograms after correction is larger than in the original interferograms, probably due to the use of global fitting parameters in the PLE5 method, indicating that the tropospheric delay phase has not been improved and, instead, erroneous signals have been introduced. Therefore, careful consideration must be given to the selection of the tropospheric delay correction method, as the deformation results in the study area may be compromised if an unsuitable correction method is used.

The mean phase standard deviation is 3.578 rad, with a reduction of approximately 8.6%. After correction by the PLE5 model, it decreases to 3.414 rad, a reduction of approximately 12.8% (see Table 2). Therefore, careful consideration must be given to the selection of the tropospheric delay correction method, as inappropriate methods may affect the deformation results in the study area.

All the results demonstrate that the mean phase standard deviation of the interferograms corrected by the PLE5 model is the lowest, indicating a superior correction effect on tropospheric delay compared to the other three methods. When applying the PLE5 model, it is essential to first analyze the external meteorological data to determine if a power-law relationship exists between the tropospheric delay and elevation and then use the power-law model to estimate the tropospheric delay. The experiments in this study reveal that, while the PLE5 model outperforms the traditional linear model in phase-based correction, it is significantly slower in terms of computational efficiency. Consequently, selecting an appropriate method for tropospheric delay estimation is crucial when performing corrections in different study areas.

## 5. Discussion

This study explored the integration of ERA-5 reanalysis data and a power-law model for InSAR tropospheric delay correction, which effectively estimates spatially varying atmospheric signals in interferometric phases [24]. Compared to correction methods based on the ERA-Interim dataset, ERA-5 delivers improved performance in mitigating tropospheric delays, yielding reliable results [29]. This integration demonstrates significant improvements in correcting tropospheric delays in InSAR applications.

To further evaluate its performance, the PLE5 model was compared with three traditional correction methods: the linear model, the ERA-5 numeric weather model, and the GACOS model. The results indicate that the PLE5 model achieves lower mean phase standard deviation in corrected interferograms and reduces correlations between elevation and delay more effectively than the other methods. In the numeric weather model by ERA-5 dataset, these findings highlight its reliability in addressing complex atmospheric conditions and its potential to improve tropospheric delay correction accuracy.

The empirical linear model establishes a simplified functional relationship between phase and elevation, which limits its effectiveness in correcting for turbulent mixing delays that are independent of elevation. Similarly, while the GACOS model is user-friendly, probably because of the complicated terrain conditions of Dali, it sometimes struggles to capture the complex atmospheric variability. Single power-law models, which rely solely on sounding balloon data, are constrained by their sparse spatial coverage, thereby restricting their applicability in regions with insufficient data density. By contrast, the PLE5 model accounts for atmospheric heterogeneity, making it well suited to regions with significant spatial variability. Despite its demonstrated effectiveness in reducing atmospheric noise, several limitations emerged during implementation.

The results of InSAR tropospheric delay correction indicate that the PLE5 model retains some uncertainties, as the correction performance for certain interferograms was not entirely satisfactory. This could be attributed to the low spatial resolution of ERA-5 data, which presents a notable challenge. ERA-5 provides meteorological data on a grid with an approximate resolution of 30 km, limiting its capacity to capture localized meteorological variations. The interpolation process cannot fully resolve the spatial heterogeneity of atmospheric conditions, leading to residual atmospheric delays in the corrected interferograms. Future work should focus on integrating ERA-5 with other ground-based meteorological observations, such as GNSS-derived water vapor data or local weather station measurements, to improve the accuracy of atmospheric water vapor interpolation and simulation. Additionally, advanced downscaling techniques could be employed to refine the spatial resolution of ERA-5 data, enabling better representation of local atmospheric variability.

In addition to spatial resolution issues, computational efficiency poses another challenge. Similar to the power-law model, the proposed correction framework is computationally demanding due to its reliance on iterative fitting processes and the need to handle large volumes of InSAR data. This computational burden becomes particularly pronounced in large-scale studies or near-real-time monitoring scenarios. Addressing this limitation will require algorithmic optimization, such as reducing the number of iterations or leveraging high-performance computing techniques. Developing computationally efficient models will be essential for expanding the applicability of the proposed method in operational deformation monitoring tasks.

With advancements in observational technologies and numerical weather models, the precision and resolution of water vapor observations are expected to improve significantly. These developments will enable more accurate and localized corrections of InSAR atmospheric errors. Integrating multi-source water vapor data—such as GNSS-derived observations, ground-based meteorological measurements, and satellite-based datasets—with advanced deep learning techniques offers a promising pathway for refining atmospheric correction models. Additionally, as observation systems and correction methods continue to evolve, standardized InSAR atmospheric correction products, akin to precise orbit data, could be made directly available. This would greatly reduce the complexity of applying atmospheric corrections, lower technical barriers, and significantly enhance the quality and reliability of InSAR deformation results.

## 6. Conclusions

This study proposed a tropospheric delay correction method (PLE5) that integrates ERA-5 reanalysis data into the power-law model to address the limitations of InSAR atmospheric corrections in regions with sparse sounding balloon observations. The method was validated using Sentinel-1 data over the Dali highland basin and compared against the GACOS model, the traditional linear model, and ERA-5-based correction. The results demonstrate that the PLE5 model achieved the lowest phase standard deviation among the tested methods, effectively mitigating atmospheric phase delay in InSAR time-series analysis.

The integration of ERA-5 data provides a practical alternative to sounding balloon observations, enabling the application of the power-law model in data-scarce regions. However, this study highlights certain challenges, including the low spatial resolution of ERA-5 data, which limits its ability to capture localized atmospheric variations. Furthermore, the computational demands of the power-law model and its susceptibility to phase outliers in certain interferograms underscore the need for further refinement.

The results demonstrate that the PLE5 method effectively enhances InSAR tropospheric delay correction by integrating ERA-5 data into the power-law model, offering a practical alternative in regions lacking sounding balloon observations. These findings serve as a reference for future research into InSAR atmospheric delay correction studies over complex terrains.

## Figures and Tables

**Table 1:** Interferometric pair baseline information.

Interferometric Pair (yyyymmdd)	Perpendicular Baseline (m)	Temporal Baseline (day)	Mean Coherence
20190519/20190624	−19	−36	0.40
20201016/20201109	−61	−24	0.48
20201109/20201203	−15	−24	0.17
20210426/20210601	−36	−36	0.60

**Table 2:** Mean value of phase standard deviation after correction by four methods.

	Original Interferogram	GACOS Correction	Linear Model Correction	ERA5 Correction	PLE5 Model Correction
Phase (rad)	3.915	3.639	3.581	3.578	3.414

## Data Availability

Data are contained within the article.
